# Dogs are sensitive to small variations of the Earth’s magnetic field

**DOI:** 10.1186/1742-9994-10-80

**Published:** 2013-12-27

**Authors:** Vlastimil Hart, Petra Nováková, Erich Pascal Malkemper, Sabine Begall, Vladimír Hanzal, Miloš Ježek, Tomáš Kušta, Veronika Němcová, Jana Adámková, Kateřina Benediktová, Jaroslav Červený, Hynek Burda

**Affiliations:** 1 Department of Game Management and Wildlife Biology, Faculty of Forestry and Wood Sciences, Czech University of Life Sciences, 16521 Praha 6, Czech Republic; 2 Department of General Zoology, Faculty of Biology, University of Duisburg-Essen, 45117 Essen, Germany

**Keywords:** Magnetoreception, Magnetosensitivity, Magnetic field, Magnetic storm, Magnetic alignment, Dog, Canid, Mammal

## Abstract

**Introduction:**

Several mammalian species spontaneously align their body axis with respect to the Earth’s magnetic field (MF) lines in diverse behavioral contexts. Magnetic alignment is a suitable paradigm to scan for the occurrence of magnetosensitivity across animal taxa with the heuristic potential to contribute to the understanding of the mechanism of magnetoreception and identify further functions of magnetosensation apart from navigation. With this in mind we searched for signs of magnetic alignment in dogs. We measured the direction of the body axis in 70 dogs of 37 breeds during defecation (1,893 observations) and urination (5,582 observations) over a two-year period. After complete sampling, we sorted the data according to the geomagnetic conditions prevailing during the respective sampling periods. Relative declination and intensity changes of the MF during the respective dog walks were calculated from daily magnetograms. Directional preferences of dogs under different MF conditions were analyzed and tested by means of circular statistics.

**Results:**

Dogs preferred to excrete with the body being aligned along the North–South axis under calm MF conditions. This directional behavior was abolished under unstable MF. The best predictor of the behavioral switch was the rate of change in declination, i.e., polar orientation of the MF.

**Conclusions:**

It is for the first time that (a) magnetic sensitivity was proved in dogs, (b) a measurable, predictable behavioral reaction upon natural MF fluctuations could be unambiguously proven in a mammal, and (c) high sensitivity to small changes in polarity, rather than in intensity, of MF was identified as biologically meaningful. Our findings open new horizons in magnetoreception research. Since the MF is calm in only about 20% of the daylight period, our findings might provide an explanation why many magnetoreception experiments were hardly replicable and why directional values of records in diverse observations are frequently compromised by scatter.

## Introduction

Magnetic alignment, i.e., spontaneous alignment of the body with respect to the magnetic field lines, when other determinants (e.g. wind direction, sun position, curiosity) of the body position are negligible, has been demonstrated in several species of mammals in diverse behavioral contexts: in grazing and resting cattle, roe deer and red deer [1-4] and hunting red foxes [5] as well as in several other mammalian species (under preparation). Magnetic alignment proved to be a suitable paradigm to scan for the occurrence of magnetosensitivity across animal taxa with a heuristic potential to contribute to the understanding of the mechanism of magnetoreception and identify further functions of a magnetic sense apart from navigation [1-9]. With this in mind we decided to look for examples of expression of magnetic alignment in dogs. Expecting magnetoreception in dogs is reasonable given the extraordinary homing abilities of dogs [10] and closely related species like red foxes, coyotes and grey wolves [11-13]. Wolves, as the progenitors of domestic dogs, possess extraordinary large (about 150–200 km^2^) home ranges [14], suggesting superior orientation abilities. Furthermore, strong indications for magnetosensation in the red fox [5] add on to the growing evidence.

A discovery of magnetoreception in dogs would open totally new horizons for magnetobiological research: Dogs are widely available experimental subjects all over the world and can easily be trained to react on diverse sensory stimuli [15]. In addition, as dogs are still readily used as experimental animals in a wide array of biomedical applications [16], the discovery of a new sense would have far reaching consequences also in this sector.

Having been inspired by our hitherto observations in other animals [1-3,5-8,17], we monitored spontaneous alignment in dogs during diverse activities (resting, feeding and excreting) and eventually focused on excreting (defecation and urination incl. marking) as this activity appeared to be most promising with regard to obtaining large sets of data independent of time and space, and at the same time it seems to be least prone to be affected by the surroundings.

## Results

**Figure 1:**
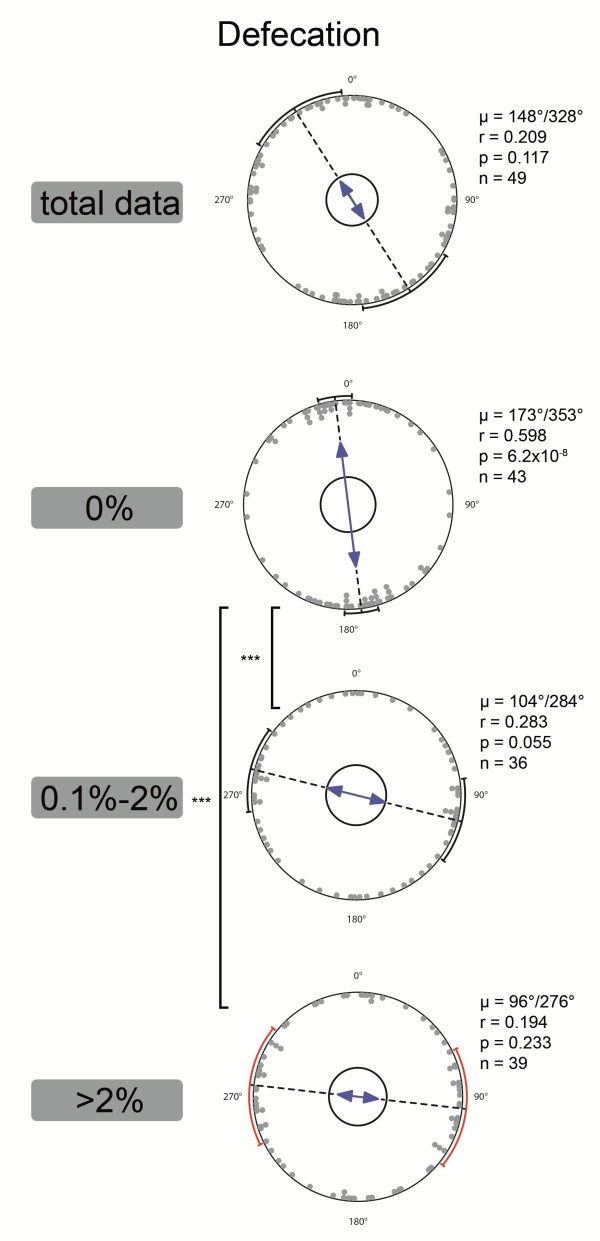
**Analysis of dog body alignment during defecation.** Axial analysis of mean vectors of dogs with more than 5 observations. Total data and observations from three different categories of relative changes of the declination of the Earth’s magnetic field are shown from top to bottom (0%, 0.1-2%, >2%).Each pair of opposite dots indicates the axis of the mean vector of all observations of a single dog. The direction (μ) and length (r) of the (grand) mean vector and the p-value of the Rayleigh uniformity test as well as the sample size are given next to each diagram. μ and r are indicated by the direction and length of the blue arrows, respectively. Small inner circles indicate the 5%-significance level of the Rayleigh test. Circle segments at the outer circle represent the 95%-confidence intervals (red circle segments indicate intervals that could not be calculated with confidence due to large circular standard deviations). Statistically significant differences between the distributions according to the Mardia-Watson-Wheeler test are indicated by asterisks (*** = p < 0.001). A significant N-S axial orientation (i.e., 95%-confidence interval includes the N-S axis) can only be seen under conditions of zero declination change. See Tables 1–2 for further details on statistics.

Circular analysis of the distribution of all values of all dogs irrespective of the magnetic field conditions revealed significant but highly scattered axial orientation during defecation (Table 1). This orientation was, however, not confirmed by the grand mean vector (calculated over the dogs’ mean values, Figure 1, Table 1). Since no significant differences between males and females and since no angular preferences during defecation were found (not shown here), we only present the axial analyses combined for both sexes here.

**Table 1:** Analysis of body orientation during defecation (all records, i.e., no differentiation between different magnetic conditions)

	**Defecation: all records**			
**Variable**	**Pooled**	**Pooled**	**Means (n > 5)**	**Means (n > 5)**
Data type	angular	axial	angular	axial
Number of observations	1,893	1,893	49	49
Mean vector (μ)	133°	157°/337°	80°	148°/328°
Length of mean vector (r)	0.013	0.052	0.043	0.209
Circular standard deviation	169°	70°	144°	51°
95% Confidence interval (-/+) for μ	-	-	-	121°-174°
99% Confidence interval (-/+) for μ	-	-	-	112°-183°
Rayleigh test (Z)	0.307	5.203	0.091	2.143
Rayleigh test (p)	0.736	0.006	0.914	0.117

**Figure 2:**
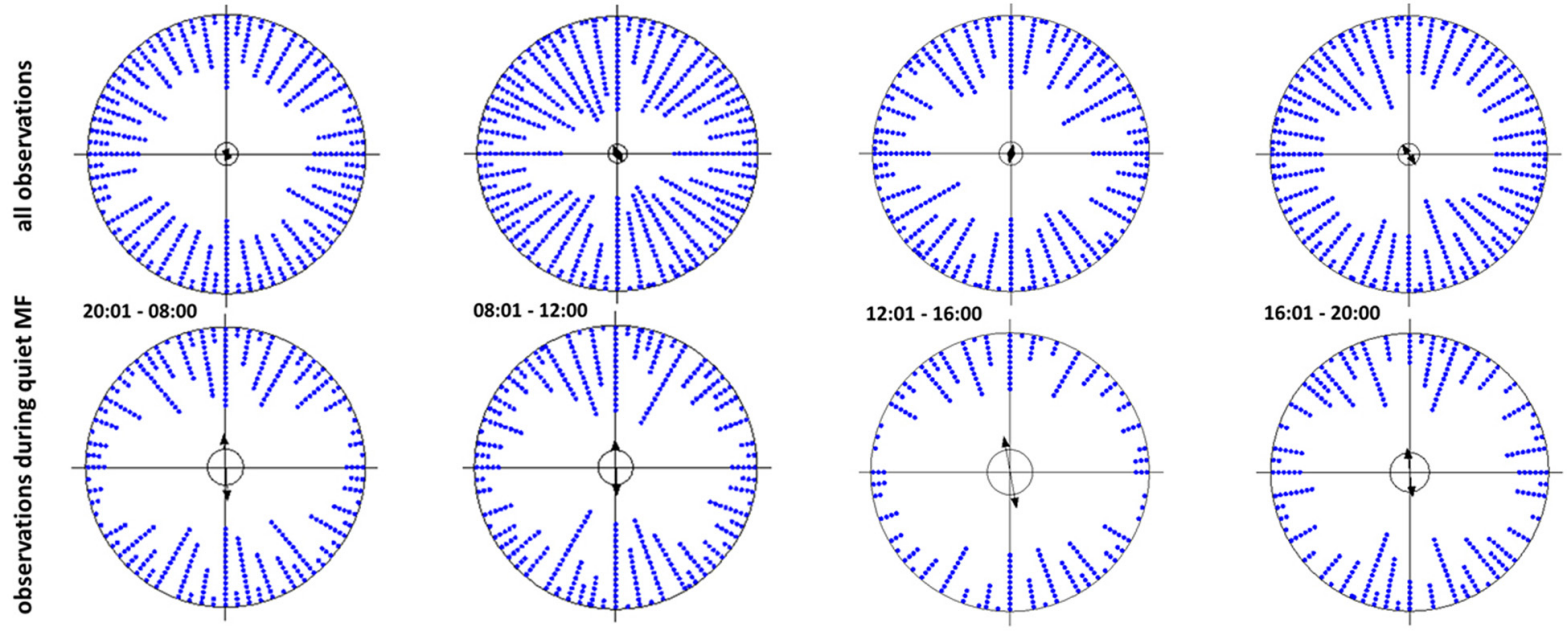
**Alignment during defecation in dogs (females and males) in different day periods.** Columns denoted “quiet MF (magnetic field)” give statistic values based on analysis of those data from the respective column “all values” which were collected under conditions of stable declination (0% change). Due to small sample sizes for single dogs, the data of all observations done in the given period are pooled. Pooling is justified in this case because samples for respective dogs have comparable sizes and because males and females show comparable posture and alignment during defecation. The data were not sorted here according to months but the distribution of observations during respective months of the year is comparable, so that the distribution of the data (and resulting analysis) would not change if winter and summer observations were further separated. Note random circular distribution of the alignment when all data for respective time periods are analyzed, but highly significant preference for the North–South axis when only observations made under quiet magnetic field are considered.

After the data were sorted according to the magnetic field conditions (specifically, Kp-index, relative changes of magnetic field intensity or of declination) at the time of recording, a differentiated picture emerged. The relative declination change proved to be the best predictor of alignment, i.e.,. sorting the data according to this parameter provided the most significant results. Analysis of pooled recordings as well as of mean vectors of recordings in dogs sampled during calm magnetic field conditions (relative change in declination = 0%; minimum of 5 observations per dog) revealed a highly significant axial preference for North–South alignment during defecation (for 0% declination change: μ = 173°/353° ± 9° (mean vector orientation angle; 95% confidence interval), r = 0.598 (mean vector length), Rayleigh test: n = 43, p = 6.2⋅10^-8^, Z = 15.353; second order (weighted) statistics: weighted mean vector (WMV): 175°/355°, r = 0.253, Hotelling test: n = 43, p = 1.02⋅10^-7^, F = 24.463; Tables 2 and 3, Figures 1 and 2). With increasing relative declination changes the distribution of dogs’ body orientations became more scattered and in the category “> 2%” the distribution was random, and no directional preference was apparent (Table 2, Figure 1). The distributions of dogs’ body orientations in the intervals of relative declination change “0.1%-2%” as well as “> 2%” were significantly different from the distribution at 0%, both, when pooled raw data and when means per dog were analyzed (Mardia-Watson-Wheeler test, p < 0.001, Figure 1). The same dependence of the directional preference on the relative changes of the magnetic declination appeared when males and females were treated separately (not shown here).

**Table 2:** Axial analysis of alignment during defecation in all dogs (pooled data or mean vectors of particular dogs sorted into three categories according to the rate of changes of magnetic field declination)

	**Pooled raw data**			**Means per dog (n > 5)**		
**Declination rate**	**0%**	**0.1-2%**	**>2%**	**0%**	**0.1-2%**	**>2%**
Number of observations	607	542	744	43	36	39
Mean vector (μ)	176°/356°	111°/291°	109°/289°	173°/353°	104°/184°	96°/276°
Length of mean vector (r)	0.216	0.106	0.03	0.598	0.283	0.194
Circular standard deviation	50°	61°	76°	29°	45°	52°
95% Confidence interval (-/+) for μ	168°-183°	-	-	164°-182°	81°-127°	-
99% Confidence interval (-/+) for μ	166°-185°	-	-	161°-185°	74°-134°	-
Rayleigh test (Z)	28.248	6.133	0.672	15.353	2.891	1.464
Rayleigh test (p)	<10^-12^	0.002	0.511	6.20⋅10^-8^	0.055	0.233

**Table 3:** Alignment during defecation in dogs (females and males) in two-hour periods

**Variable**	**All records 20:01–08:00**	**Quiet MF 20:01–08:00**	**All records 08:01–12:00**	**Quiet MF 08:01–12:00**	**All records 12:01–16:00**	**Quiet MF 12:01–16:00**	**All records 16:01–20:00**	**Quiet MF 16:01–20:00**
Number of observations	442	173	599	188	396	109	455	144
Mean vector (μ)	157°/337°	177°/357°	152°/332°	178°/358°	12°/192°	170°/350°	147°/327°	173°/353°
Length of mean vector (r)	0.042	0.239	0.073	0.2	0.074	0.26	0.088	0.176
Circular standard deviation	72°	48°	65°	51°	65°	47°	63°	53°
95% Confidence interval (-/+) for μ	112°-202°	165°-190°	130°-174°	163°-192°	345°-39°	155°-184°	126°-168°	155°-192°
99% Confidence interval (-/+) for μ	98°-217°	161°-193°	123°-181°	159°-106°	337°-48°	151°-189°	119°-175°	149°-198°
Rayleigh test (Z)	0.769	9.866	3.17	7.533	2.145	7.367	3.54	4.474
Rayleigh test (p)	0.463	0.000052	0.042	0.000535	0.117	0.000632	0.029	0.011

Columns denoted “quiet MF (magnetic field)” give statistic values based on analysis of those data from the respective column “all values” which were collected under conditions of stable declination (0% change). Due to small sample sizes for single dogs, the data of all observations done in the given period are pooled. Pooling is justified in this case because samples for respective dogs have comparable sizes and because males and females show comparable posture and alignment during defecation. The data were not sorted here according to months but the distribution of observations during respective months of the year is comparable, so that the distribution of the data (and resulting analysis) would not change if winter and summer observations were further separated. Note random circular distribution of the alignment when all data for respective time periods are analyzed, but highly significant preference for the North–South axis when only observations made under quiet magnetic field are considered.

Analysis of the alignment during defecation under conditions of stable magnetic declination (0% changes) revealed no significant effect of sex. There may be a slight effect of age: dogs in the age category 2.5-7 years showed a clearer preference than younger or elder dogs (not shown). The effect of the dog breed could not be tested because of small sample sizes.

**Figure 3:**
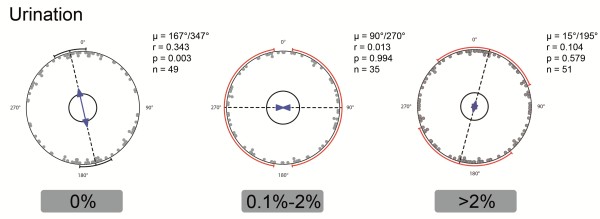
**Analysis of dog body alignment during urination.** Axial analysis of mean vectors of dogs of both sexes with at least five observations. Observations from three different categories of relative changes of the declination of the Earth’s magnetic field are shown from left to right (0%, 0.1-2%, >2%). Each pair of opposite dots indicates the axis of the mean vector of all observations of a single dog. The direction (μ) and length (r) of the (grand) mean vector and the p-value of the Rayleigh uniformity test as well as the sample size are given next to each diagram. μ and r are indicated by the direction and length of the blue arrows, respectively. Small inner circles indicate the 5%-significance level of the Rayleigh test. Circle segments at the outer circle represent the 95%-confidence intervals (red circle segments indicate intervals that could not be calculated with confidence due to large circular standard deviations). A significant N-S axial orientation (i.e., 95%-confidence interval includes the N-S axis) can only be seen under conditions of zero declination change. See Tables for further details on statistics.

Circular analysis of the distribution of the pooled raw data demonstrated a significant deviation from random distribution also in urinating dogs (Table 4). Analyzing this data for males and females separately we found a slight difference in the patterns between sexes: Pooled data (without the dog M07) and mean values of all males with at least 5 observations revealed a significant angular preference for North-West heading during urination (Table 5). The male borzoi M07 contributed approximately one third of the urination data and was analyzed separately (Table 6); the results were similar to the pooled data of all other males. In contrast, females showed an axial preference for approximately the North–South axis during urination (Table 7). As in the case of defecation, sorting the data according to the relative changes of declination revealed a significant effect of this factor and a significant axial North–South alignment only under calm MF conditions (for 0% declination change: μ = 167°/347° ± 16°, r = 0.343, Rayleigh test: n = 49, p = 0.003, Z = 5.766; second order (weighted) statistics: WMV: 173°/353°, r = 0.165, Hotelling test: n = 49, p = 5.08⋅10^-4^, F = 8.952; Figure 3, Tables 5, 6, 7). The raw data distributions during changing declination were significantly different from the distribution under calm magnetic conditions (Mardia-Watson-Wheeler test, p < 0.05, Figure 3).

**Table 4:** Angular and axial analysis of body orientation in dogs during urination

	**Urination: (all records)**									
**Data type**	**Angular**					**Axial**				
	**Pooled**			**Means (n ≥ 5)**		**Pooled**			**Means (n ≥ 5)**	
	**Males**	**M07**	**Females**	**Males**	**Females**	**Males**	**M07**	**Females**	**Males**	**Females**
Number of observations	1,402	2,478	1,702	24	35	1,402	2,478	1,702	24	35
Mean vector (μ)	312°	298°	13°	292°	331°	154°/334°	175°/355°	5°/185°	89°/269°	11°/191°
Length of mean vector (r)	0.048	0.105	0.03	0.213	0.213	0.037	0.196	0.132	0.101	0.292
Circular standard deviation	141°	122°	152°	101°	101°	74°	52°	58°	61°	45°
95% Confidence interval (-/+) for μ	268°-356°	283°-313°	309°-78°	217°-8°	269°-34°	125°-183°	171°-179°	358°-12°	9°-169°	348°-33°
99% Confidence interval (-/+) for μ	254°-10°	278°-318°	288°-98°	193°-31°	249°-53°	116°-192°	169°-180°	356°-15°	343°-194°	341°-40°
Rayleigh test (Z)	3.215	27.075	1.517	1.088	1.584	1.875	94.735	29.524	0.246	2.99
Rayleigh test (p)	0.04	1.74⋅10^-12^	0.219	0.341	0.206	0.153	< 10^-12^	< 10^-12^	0.786	0.049
Rao’s spacing test (U)	307.618	354.479	339.271	140.383	131.675	325.078	357.094	346.675	134.75	145.847
Rao’s spacing test (p)	<0.01	<0.01	<0.01	>0.1	>0.1	<0.01	<0.01	<0.01	>0.1	>0.1

Data for the male dog M07 are presented in a separate column due to large sample size.

**Table 5:** Angular analysis of alignment during urination in all males (pooled data without dog M07 and mean vectors of all males sorted into three categories according to the relative changes of magnetic field declination)

	**Pooled raw data**			**Means per dog (n ≥ 5)**		
**Declination rate**	**0%**	**0.1-2%**	**>2%**	**0%**	**0.1-2%**	**>2%**
Number of observations	491	256	655	22	15	22
Mean vector (μ)	293°	12°	84°	291°	355°	195°
Length of mean vector (r)	0.129	0.08	0.006	0.367	0.349	0.07°
Circular standard deviation	116°	129°	182°	81°	83°	132°
95% Conf. interval (-/+) for μ	265°-321°	310°-74°	-	246°-335°	290°-61°	315°-76°
99% Conf. interval (-/+) for μ	256°-329°	290°-94°	-	232°-349°	270°-81°	239°-151°
Rayleigh test (Z)	8.17	1.619	0.028	2.959	1.828	0.109
Rayleigh test (p)	2.83⋅10^-4^	0.198	0.973	0.05	0.162	0.899
Rao’s spacing test (U)	260.285	281.656	283.053	148.258	147.139	127.188
Rao’s spacing test (p)	<0.01	<0.01	<0.01	>0.1	>0.1	>0.5

**Table 6:** Angular analysis of alignment during urination in male borzoi (M07)

**Declination rate**	**0-1.7%**	**1.8-3.3%**	**≥3.4%**
Number of observations	957	818	703
Mean vector (μ)	310°	285°	280°
Length of mean vector (r)	0.154	0.08	0.078
Circular standard deviation	111°	129°	130°
95% Confidence interval (-/+) for μ	294°-327°	250°-320°	241°-318°
99% Confidence interval (-/+) for μ	289°-332°	240°-331°	229°-331°
Rayleigh test (Z)	22.64	5.18	5.517
Rayleigh test (p)	1.47⋅10^-10^	0.006	0.015
Rao’s spacing test (U)	345.705	344.156	341.565
Rao’s spacing test (p)	< 0.01	< 0.01	< 0.01

Data are sorted into three categories according to the *relative changes* of magnetic field declination. Limits of the categories were chosen so that sample sizes are comparable.

**Table 7:** Axial analysis of alignment during urination in all females (pooled data and mean vectors of particular dogs sorted into three categories according to the relative changes of magnetic field declination)

	**Pooled raw data**			**Means per dog (n ≥ 5)**		
**Declination rate**	**0%**	**0.1-2%**	**>2%**	**0%**	**0.1-2%**	**>2%**
Number of observations	603	396	703	27	20	29
Mean vector (μ)	5°/185°	2°/182°	11°/191°	0°/180°	7°/187°	23°/203°
Length of mean vector (r)	0.208	0.131	0.068	0.434	0.159	0.134
Circular standard deviation	51°	58°	66°	37°	55°	57°
95% Conf. interval (-/+) for μ	357°-12°	347°-17°	349°-33°	163°-196°	312°-63°	328°-78°
99% Conf. interval (-/+) for μ	354°-15°	342°-22°	342°-40°	157°-201°	294°-80°	311°-95°
Rayleigh test (Z)	26.146	6.839	3.251	5.085	0.503	0.52
Rayleigh test (p)	4.41⋅10^-12^	0.001	0.039	0.005	0.61	0.598
Rao’s spacing test (U)	312.836	330	339.004	156.975	135.002	129.554
Rao’s spacing test (p)	<0.01	<0.01	<0.01	>0.05	>0.1	>0.5

## Discussion

Dogs rely much on their owner, and for many tasks they might anticipate the demanded behavior by reading their owner’s facial expression and make use of unintentional experimenter-given cues [18-20]. This adds a bias-trap to any research relying on behavioral studies and particularly conditioning. However, this is certainly not a confounder in our study because the dogs do not have to fulfill a certain task, but perform everyday routine behavior.

The study was truly blind. Although the observers were acquainted with our previous studies on magnetic alignment in animals and could have consciously or unconsciously biased the results, no one, not even the coordinators of the study, hypothesized that expression of alignment could have been affected by the geomagnetic situation, and particularly by such subtle changes of the magnetic declination. The idea leading to the discovery of the correlation emerged after sampling was closed and the first statistical analyses (with rather negative results, cf. Figure 1) had been performed. Also, the acquisition of data on magnetic declination was carried out without knowledge of heading values on the respective time and date.

We found no differences in alignment of females and males during defecation and of females during urination, which might be related to a similar posture the animals are adopting during defecation (in all dogs) and urination (in females). Urinating males have a slightly different preference to orient their body axis than urinating females (cf. Figure 3); this could be caused by leg lifting during urination in males. Indications of different directional tendencies depending on which leg (left or right) is lifted are currently under study. All recordings were made outside on open fields, and routes of walks were routinely changed to exclude or limit pseudoreplications which would arise when dogs are defecating or urinating at just a few places within their kennel or house yard.

Natural fluctuations of the Earth’s magnetic field [21,22] have previously been suggested to disturb orientation in birds [23-25], bees [26] and whales [27]; and even to affect vegetative functions and behavior in humans [28,29], reviewed also in [22].

In this study, we provide the first clear and simply measurable evidence for influence of geomagnetic field variations on mammal behavior. Furthermore, it is the first demonstration of the effect of the shift of declination, which has to our knowledge never been investigated before. Previous studies of the effect focused mainly on the variations in field intensity. Although intensity and declination changes are mostly concomitant, declination change was a better predictor of dog alignment. Interestingly, the rate and direction of the changes disturb more effectively than absolute values. Here, for the first time the response can be attributed to the rate of magnetic field changes.

Typically, the daily declination comprises westward-shifts in the morning and eastward-shifts in the afternoon, while the magnetic field is rather stable at night [21,22]. This calls for necessity to test whether the dog alignment is not actually influenced primarily by time of the day and most probably by position of the sun on the sky. We can, however, exclude this alternative. First, days when the magnetic field parameters change erratically and unpredictably (i.e., magnetic storms) are quite frequent. These changes have been well studied by others and are described in the literature (cf. [21,22] for reviews). Second, the data collection was not biased to either morning or afternoon (Table 8). Third, periods of sampling under conditions of quiet magnetic field were rather evenly distributed in the course of the day. Fourth, and most importantly, alignment during excreting was apparent under conditions of quiet magnet field, irrespective of the time of day or month. Time of day per se was not a reliable predictor of expression of alignment (Figure 2, Tables 3, 9). Fifth, generally, there are on average 1,450 sunshine hours per year at maximum in the Czech Republic and in Germany, on localities where measurements were done. Even if we would assume that these sunshine hours were evenly distributed over the daylight period and the year (as our observations were), there would only be a probability of 33% that the observation was made when the sun was visible. Hence, with high probability (67%) most walks during the daylight period were made when it was cloudy.

**Table 8:** Proportion of observations made under different conditions of the Earth’s magnetic field expressed in rate of changes of declination during the sampling period

**Declination changes (%)**	**Proportion of observations (%)**
0	18
0.1-1.0	6
1.1-2.0	19
2.1-3.0	17
3.1-4.0	16
4.1-5.0	12
5.1-6	3
6.1-8.0	6
>8.1	3

**Table 9:** Proportion of measurements of alignment sampled during 2 hrs-periods (and during the night) and proportion of measurements (from the total) sampled in respective periods under conditions of quiet magnetic field (i.e., with no changes in declination)

**Period (time)**	**Proportion of all observations (%)**	**Proportion of observations under quiet MF (%)**
05:01–07:00	2.2	1.6
07:01–09:00	9.9	12.2
09:01–11:00	26	18.5
11:01–13:00	16.8	6.3
13:01–15:00	11.5	12.8
15:01–17:00	13.6	20.8
17:01–19:00	10.3	5.8
19:01–21:00	7.9	18
21:01–23:00	1.3	2.8
23:01–05:00	0.5	1.2

Last but not least, the argument that the dogs might orient with regard to sun position so that they turn with their back to the sun in order to avoid dazzling by sunshine during such a sensitive and vulnerable act as excretion can be questioned. This argument is not plausible for urine marking, which is a brief act. We doubt that a dog that cares of not being attacked would always make sure to be turned away from the sun. The dog will likely look in that direction from where danger can most probably be expected - and this is for sure not always the direction away from the sun. In contrast to a human, the dog is relying also on its nose and its ears (in some breeds even more than on its eyes) when monitoring its surroundings - so we may expect that the dog heads with its nose and pinnae against the wind or in the direction of interest. Directing the pinnae and the nose may take priority over eyes. One can also often observe that dogs (especially during defecation) align in a certain direction, which is actually a different one from the direction of interest and they turn their head then in that other direction. Also we have to take into account that dogs are smaller than humans, they look at a different angle over the horizon and even in situations when we are dazzled, they might be not. Quite important: note also that the preference is axial - there are many cases when the dog actually looks southwards. There is no evidence for shift of the alignment axis during the day.

It is still enigmatic why the dogs do align at all, whether they do it “consciously” (i.e., whether the magnetic field is sensorial perceived (the dogs “see”, “hear” or “smell” the compass direction or perceive it as a haptic stimulus) or whether its reception is controlled on the vegetative level (they “feel better/more comfortable or worse/less comfortable” in a certain direction). Our analysis of the raw data (not shown here) indicates that dogs not only *prefer* N-S direction, but at the same time they also *avoid* E-W direction. The fact that larger and faster changes in magnetic conditions result in random distribution of body directions, i.e., a lowering of the preferences and ceasing of the avoidances, can be explained either through disturbing or conscious “shutdown” of the magnetoreception mechanism. From the two putative mechanisms that are discussed in birds and other vertebrates (radical-pairs and single-domain or superparamagnetic particles [30,31]) both might account for the observed alignment of the dogs and their sensitivity to declination changes.

An answer may lie in the biological meaning of the behavior: if dogs would use a visual (radical-pair based) magnetic map to aid general orientation in space as has been proposed for rodents [32], they might have the need to center/calibrate the map now and then with regard to landmarks or a magnetic reference. Aligning the map and the view towards North (or South) facilitates reading the map. Furthermore, calibration only makes sense when the reference is stable and reliable. We might think of this the same way as a human is stopping during a hike to read a map. When the map is blurred or the reference (perceived magnetic direction) is dispersed or moving due to magnetic disturbances, however, calibration is impossible. In the case of the dogs it thus would totally make sense to not pay attention to magnetic body alignment any more under conditions of a shifting magnetic field.

## Conclusions

We demonstrate, for the first time (a) magnetic sensitivity in dogs, (b) a measurable, predictable behavioral reaction upon natural magnetic field (MF) fluctuation in a mammal, and (c) high sensitivity to small changes in polarity, rather than in intensity, of the MF. Our findings open new horizons in magnetoreception research. The newly introduced animal model (dog), paradigm (alignment during excretion) and parameter (relative declination change) open new horizons for biomagnetic research. Particularly the findings that already small fluctuations in Earth’s magnetic field elicit a behavioral response and the fact that “normal” magnetic conditions under which dogs express their orientation behavior occur only in about 30% of all cases call for caution. When extrapolated upon other animals and other experiments and observations on animal magnetoreception, this might explain the non-replicability of many findings and high scatter in others. Behavioral scientists need to revise their former experiments and observations and consider the phenomenon in their current and future experiments. The phenomenon challenges biophysicists to formulate testable hypotheses for mechanisms responsible for magnetoreception of inconsistencies of the direction of the MF direction. Finally, it forces biologists and physicians to seriously reconsider effects magnetic storms might pose on organisms.

## Methods

**Figure 4:**
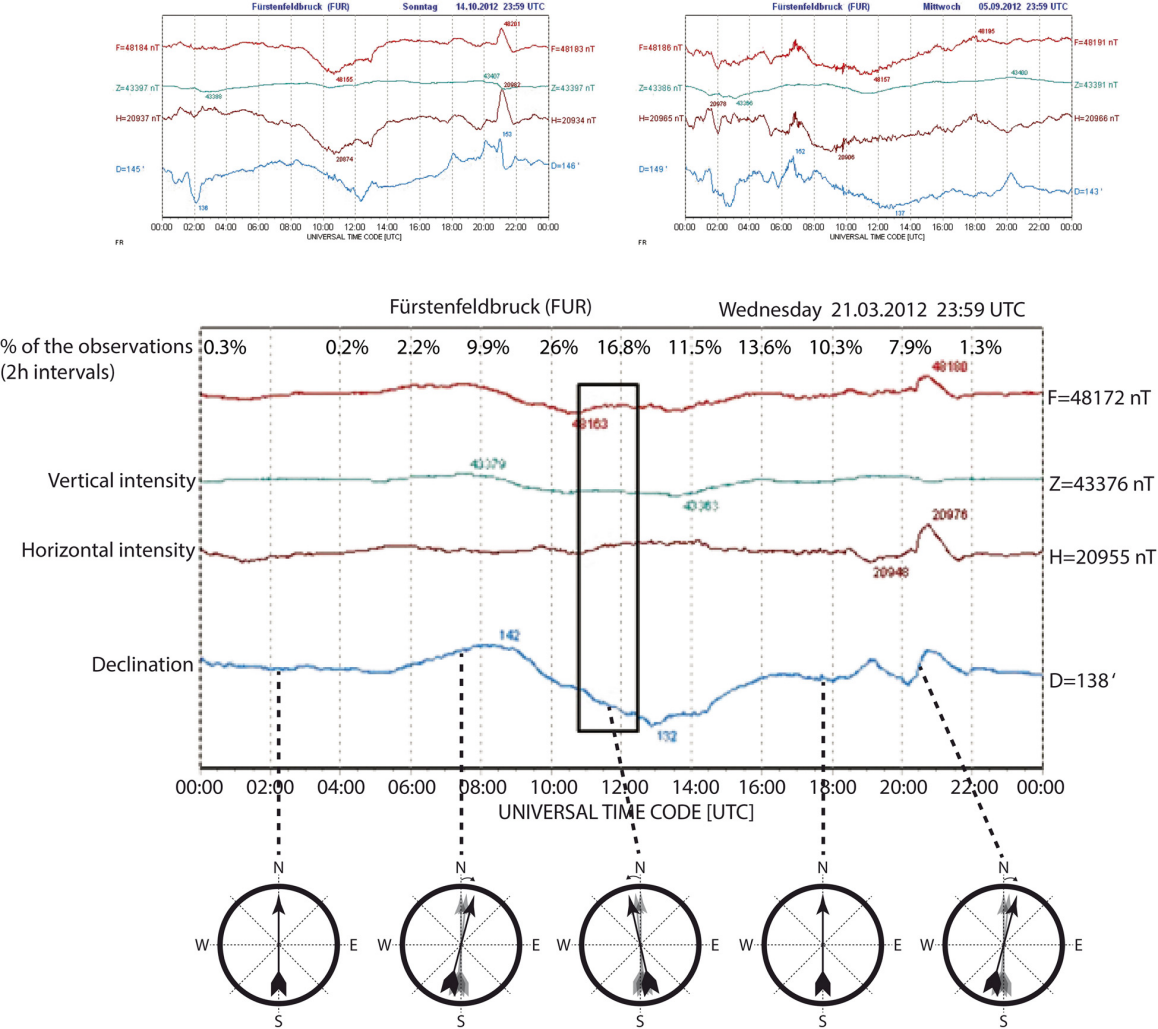
**An example of three typical daily magnetograms obtained from the Geomagnetic Observatory Fürstenfeldbruck (Munich, Germany): **http://www.geophysik.uni-muenchen.de/observatory/geomagnetism **F = total intensity, Z = vertical intensity, H = horizontal intensity, D = declination of the Earth’s magnetic field.** In the given time period (marked by a rectangle in the lower graph), declination was changing westwards with the following rate: from 142 to 132 arch minutes in 240 time minutes = Difference of 10 arch minutes/240 min. = 4.2 %. The row of compasses illustrates the effect of the declination change in a highly exaggerated manner at different times of the day shown in the example. In reality, the changes in MF direction were much smaller. Note that even though the daily declination changes show some regularity (cf. ref. 22) they are not reliably predictable as illustrated by the frequent highly erratic changes, which are exemplarily shown in the two upper graphs.

Alignment of the body (along the thoracic spine) in direction towards the head (heading) was measured in freely moving dogs (i.e., not on the leash) in “open field” (on meadows, fields, in the wood etc., i.e., unconstrained, and uninfluenced by linear structures, such as walls and fences) away from the road traffic, high voltage power lines, and conspicuous steel constructions during defecation and urination by a hand-held compass (Figure 4). Dog breed, sex, age, body mass, condition, dog-ID were protocolled as well as date, time, locality, and further circumstances of recordings (e.g. within the home range, in unfamiliar surroundings etc.). Thanks to the commitment of altogether 37 dog owners/reporters and the involvement of 70 dogs (28 males, 42 females) belonging to 37 breeds (Tables 10 and 11) we collected data on heading during defecation (n = 1,893 observations; 55 dogs) and urination (n = 5,582; 59 dogs) from December 2011 till July 2013. The samples were collected in the Czech Republic and in Germany.

**Table 10:** List of recorded female dogs and respective numbers of records

	**ID dog**	**Breed**	**Reporter (Abbrev.)**	**Age (years)**	**Weight (kg)**	**n defecation**	**n urination**
1.	F22	Airedale Terrier	Hanz	4	25	39	14
2.	F06	Beagle	Nova	3	10	0	7
3.	F29	Beagle	Krej	7	10	92	52
4.	F01	Bearded Collie	Niets	11	25	102	30
5.	F23	Border Terrier	Hanz	13	7	15	2
6.	F69	Bouvier des Flandres	Elli	1	34	4	6
7.	F35	Dachshund	Hart	2.5	7	22	110
8.	F37	Dachshund	Faif	4	9	33	36
9.	F48	Dachshund	Bene	11	7	31	21
10.	F54	Dachshund	Bene	6	4	16	8
11.	F55	Dachshund	Bene	3.5	10	0	21
12.	F57	Dachshund	Bene	1	4	18	9
13.	F58	Dachshund	Bene	10	4	17	91
14.	F60	Dachshund	Bene	1	4	11	7
15.	F82	Dachshund	Bene	5.5	6.5	0	0
16.	F83	Dachshund	Bene	13	6	0	0
17.	F90	Dachshund	Dohm	2	4.5	5	42
18.	F40	Dalmatian	Kriv	14	20	64	153
19.	F21	English Springer Spaniel	Hanz	1	20	58	18
20.	F81	English Springer Spaniel	Zdar	7	21	0	0
21.	F77	Entlebucher Mountain Dog	Hron	4	18	0	34
22.	F41	Fox Terrier Smooth	Adam	12	8	37	75
23.	F42	Fox Terrier Smooth	Adam	2	6.5	33	39
24.	F43	Fox Terrier Smooth	Adam	6	8.5	20	65
25.	F44	Fox Terrier Smooth	Adam	2	10	16	16
26.	F36	German Spaniel	Faif	3	13	46	33
27.	F13	German Wirehaired Pointer	Cuko	4	30	5	0
28.	F14	German Wirehaired Pointer	Cuko	7	30	3	0
29.	F20	Golden Retriever	Hanz	10	30	29	16
30.	F39	Irish Terrier	Tres	1.5	15	15	16
31.	F24	Jack Russell Terrier	Jura	3	7	0	32
32.	F75	Mix	Hron	12	20	4	79
33.	F66	Mix: Lhasa Apso/Jack Russell Terrier	Rick	4	x	22	0
34.	F09	Rhodesian Ridgeback	Nova	5	30	0	59
35.	F71	Small Münsterländer	Pali	10	24	24	50
36.	F32	Standard Schnauzer	Posp	12	6	34	125
37.	F45	Tibetan Spaniel	Hegl	6	5	14	101
38.	F70	Transylvanian Hound	Zema	0.7	30	0	10
39.	F08	Weimaraner	Nova	6	30	2	71
40.	F10	West Highland White Terrier	Nova	3	7	0	42
41.	F34	West Highland White Terrier	Hart	8	7	56	212
42.	F11	Yorkshire Terrier	Garc	6.5	2	30	0
		*records/dogs*				*917*	*1702*

**Table 11:** List of recorded male dogs and respective numbers of records

	**ID dog**	**Breed**	**Reporter (Abbrev.)**	**Age (years)**	**weight (kg)**	**n defecation**	**n urination**
1.	M27	Beagle	Krej	4	10	95	53
2.	M28	Beagle	Krej	2	10	92	53
3.	M33	Beagle	Posp	3	10	14	39
4.	M04	Bernese Mountain Dog	Leu	5	40	29	0
5.	M76	Border Terrier	Hron	7	8	0	37
6.	M07	Borzoi	Nova	4	40	96	2478
7.	M31	Coton de Tulear	Acke	4	4	11	106
8.	M05	Dachsbracke	Cerv	7	15	54	127
9.	M26	Dachshund	Komi	5	7	46	92
10.	M52	Dachshund	Bene	7	5	0	23
11.	M53	Dachshund	Bene	8	4	10	43
12.	M59	Dachshund	Bene	1	4	20	19
13.	M61	Dachshund	Bene	12	6	2	50
14.	M62	Dachshund	Bene	1	6	15	16
15.	M65	Dachshund	Faif	3	7	10	7
16.	M74	German Spitz	Hron	3.5	5	0	36
17.	M72	Hanoverian Hound	Krau	5.5	45	15	0
18.	M03	Irish Red Setter	Gros	3	30	47	0
19.	M80	Mix: German Shepherd x Schnauzer	Spor	10	35	71	85
20.	M63	Mix: Husky-Australian Shepherd	Rick	5	25	46	0
21.	M16	Norfolk Terrier	Kust	3	9	48	245
22.	M73	Norwich Terrier	Hron	3	8	0	36
23.	M46	Old English Sheepdog	Baum	4	45	38	122
24.	M19	Pug	Plac	3	9	66	60
25.	M25	Rhodesian Ridgeback	Jura	3	30	0	34
26.	M02	Schapendoes	Kour	1.5	25	86	84
27.	M30	Styrian Coarse-haired Hound	Kubi	7	15	45	19
28.	M38	Transylvanian Hound	Klem	0.5	30	20	16
		*records/dogs*				*976*	*3880*

After sampling and the first analysis (which yielded negative or at least ambiguous results) had been completed, we decided to sort the data according to the geomagnetic conditions predominating during the respective sampling times. Correlative values on Earth’s magnetic field strength and direction for all the particular times of recordings were obtained from the Geomagnetic Observatory Fürstenfeldbruck (Munich, Germany): http://www.geophysik.uni-muenchen.de/observatory/geomagnetism

Data on K and C values expressing the magnitude of disturbances in horizontal intensity of the Earth’s magnetic field were obtained from: ftp://ftp.ngdc.noaa.gov/stp/geomagnetic_data/indices/kp_ap/.

**Figure 5:**
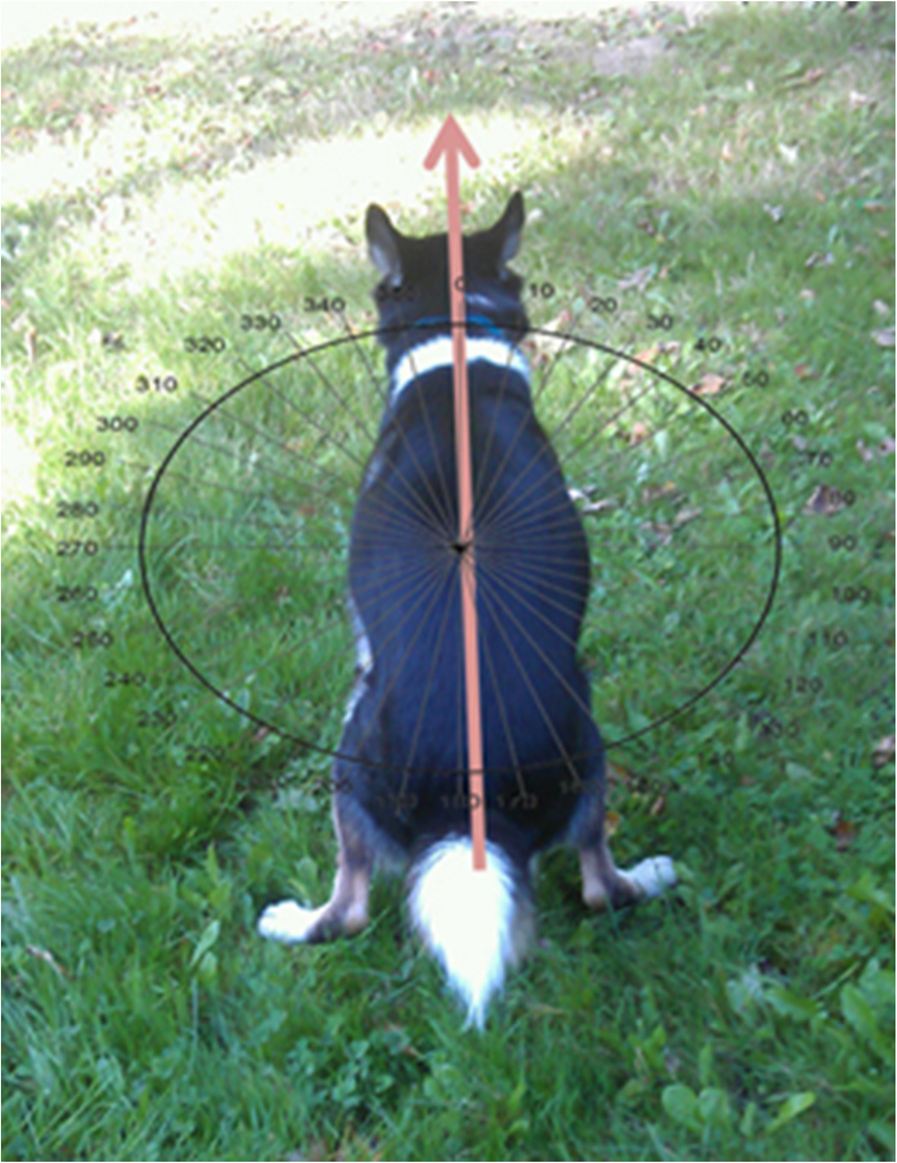
**Body orientation in dogs during defecation or urination was measured as a compass direction of the thoracic spine (between scapulae) towards the head.** (We included the photo just to illustrate the measurement. Owing to the photographer’s effort to shoot the photo with the sun from behind and to demonstrate the way of measurement, the dog on the photo looks away from the sun.) Photo Credits go to Jenny Ricken.

Relative declination and intensity changes during the respective dog walks were assorted into the categories according to the relative changes (in percent) calculated from graphs by dividing the difference between the initial and end (minimum/maximum) values by the duration (in minutes) of the respective period of changes (Figure 5).

Circular statistics were carried out with Oriana 4.02 (Kovach Computing). Both pooled individual data and means of particular dogs or walks were considered and analyzed. We performed angular and axial analysis on the measurements of each dog. Second order analysis was performed on the data which yielded the higher significance in the first order analysis (angular or axial). Only dogs with at least five measurements were analyzed. Statistically significant deviations from random distributions were investigated using the Rayleigh test of circular statistics. Differences between distributions were tested for significance with the Mardia-Watson-Wheeler test. Level of significance was set at 5%. Since about 44% data on urination under control conditions originated from one dog (M07, male borzoi) we also performed analyses for this particular dog separately.

### Ethics statement

The study did not involve any disturbance of the animals under observation.

## Competing interests

The authors declare that they have no competing interests.

## Authors’ contributions

Bur, Har, Nov conceived the study with significant input from Beg and Mal; All authors sampled data and/or coordinated sampling by assistants; Beg, Bur, Har, Mal, Nov carried out statistical analyses; Beg, Bur, Mal wrote the paper with input from Har and Nov; Bur discovered dependency of alignment upon Earth’s magnetic conditions; All authors discussed the findings; Beg, Bur, Har, Mal, Nov interpreted the observations. All authors read and approved the final manuscript.
